# Infantile Diarrhœa

**Published:** 1873-06

**Authors:** Ira E. Oatman


*Infantile Diarrhœa.* By IRA E. OATMAN, M.D. Read before the Sacramento Society for Medical Improvement.

Actuated by the prevailing incentive of this society to diversify its deliberations, and make them practical, I invite your attention to one of the most fruitful themes in medical science. The relative proportional mortality in New York, from *infantile diarrhœa*, will appear by reference to a single statistical observation: Embracing a period of five years, there were 12,293 deaths, or 2,458 per annum; the greater portion, probably, being under five years of age.

The term diarrhœa is used to represent pathological conditions of the alimentary canal, too extensive and varied to be embraced in this paper. Disregarding all nosological arrangement, I propose to treat of those cases occurring from birth to the end of the first dentition, or later, the nature of the case being the same—with due regard to their general and special pathology. The disease occurs chiefly in the hot season; this year, in Sacramento, its greatest prevalence having been in June. The delicate and impressible organism of the infant is much more susceptible to external influences than that of the adult. Whatever lowers its standard of health increases this susceptibility, in the same proportion. Some infants are feeble from birth, and require extra vigilance in regard to exposure and diet. These, and all others, have the susceptibilities of the digestive organs increased by the following agencies: the heat of summer; malarious influence; dampness, and vicissitudes of temperature; loss of sleep, fatigue, or traumatic injuries: also, teething, not *per se*, as usually taught and believed, but the slight fever, or increased nervous irritability, from the local irritation, lowers the standard of health and increases the susceptibility to the exciting causes. The same condition often results from attacks of the exanthemata, and other general and local diseases. Lastly, in towns and cities especially, noxious exhalations from the decomposition of organic matter, in alleys and back yards, but more abundantly in privies and cess-pools. These latter are among the most depressing influences in predisposing to the attack, if, indeed, they are not directly causative.

When the vitality of the delicate organism is impaired by any of the above agencies, or when it is congenitally feeble, the susceptibilities of the alimentary canal are so increased, that causes so slight as often to elude our observation are sufficient to provoke the attack.

Among the exciting causes may be mentioned the child's usual diet in excess, whether the mother's milk, or other milk, or any artificial diet. This is a very common cause. Almost any solid food may excite the attack—especially if acescent, sub-acid, or difficult to digest—as jellies, preserves, fruits, especially if not quite ripe, potatoes not finely mashed, cheese, nuts, vegetables, pickles, etc. The delicate mucous membrane of the bowels will scarcely tolerate any substance except healthy chyme, when the standard of health is in some degree lowered. Hence, the acid generated by fermentation, even of the mother's or other milk, with its undigested casein, frequently becomes an active exciting cause of diarrhœa. It is true, however, of a limited number of vigorous infants, whose digestive powers are not impaired, as above, that a large excess of proper food, or a less quantity of that more indigestible, may excite the bowels to increased action, to free themselves from the offending substance. This irritates the mucous membrane, and increases its susceptibility; and then the ordinary diet, in the usual quantities, is not digested, but acidifies, and is hurried through the bowels unchanged, devoid of the lacteal fluid, and maintains, if indeed it does not increase, that pathological condition upon which the diarrhœa depends. *The general and special pathological conditions* herein described, underlie most cases of *infantile convulsions*, which only require the agency of the exciting causes, to be fully developed.

The alimentary canal is sometimes so gorged with indigestible substances, that it is wholly unable to dispose of them, with all the increased vermicular motion which their presence excites; and they become, without manual assistance, hopelessly impacted in the rectum. I saw two cases, several years ago, both in American township, illustrative of this fact. The two cases were almost identical in all respects, except in regard to the offending substances. The children were both girls, aged about seven years. When I was called to see them, the bowels had not moved for two days—

the pain and tenesmus were frequent and extreme—the abdomen tender, somewhat tumid, and tense; the knees drawn up; the pulse small and rapid; the extremities cool and shrunken—with thirst and great restlessness. Castor oil would not operate, and the pipe of the syringe would only enter an inch or so—the fluid returning as fast as injected. The anus, in both cases, was so sensitive that the children had to be controlled by force, to enable me to examine the rectum. With the index finger, I readily detected the impacted substances, when I proceeded at once, but with difficulty, to remove them. From one rectum there came about half a pint of grape seed, so nearly dry and free from other substances, that they rattled in the tin vessel into which they were received. From the other, about the same quantity of hard, fibrous sour-crout—almost dry—which had been made from cabbage grown in summer on dry land. Under appropriate treatment, both cases recovered.

I need not allude further to the general *pathology* of the diarrhœa of infants. That is sufficiently considered in what I have read. Nor is it necessary to more than mention the special pathology, or pathological anatomy. For you all know that *diarrhœa is only a symptom* of morbid conditions of the alimentary canal, with, generally, some constitutional enervation. In one morbid state, or condition, the diarrhœa is simple—not depending upon inflammation, or, so far as we know, even congestion. The mucous membrane is found natural in appearance, or slightly reddened in some places; and in rare cases the solitary and aggregate glands are slightly enlarged. In other cases, frequently of longer standing, there is more evidence of inflammation. The mucous membrane, chiefly in the lower portion of the ilium and the sigmoid flexure of the colon, is red and thickened; both classes of glands are enlarged and elevated; the mucous membrane, and in chronic cases the solitary glands, are frequently found ulcerated. This constitutes ilio-colitis or inflammatory diarrhœa, and, when attended with pain, tenderness, tumefaction and fever, enteritis. When the inflammation is in the colon and rectum, with tormina, tenesmus, frequent mucous and bloody stools, it is dysentery. Occasionally in Sacramento, but vastly more frequently in the eastern cities of America, and, I believe in Europe, a mild case of diarrhœa will suddenly change into one with large liquid stools, rapidly increasing in frequency, resembling "rice water"—and sometimes in an hour or two becoming limpid and transparent; and usually attended with vomiting and speedy collapse. In this case, there is frequently no pathological change perceptible in the mucous membranes. I need not say that this, in America, is called cholera infantum.

In regard to the prognosis, increased quantity, fluidity, and frequency of the stools are unfavorable, especially if attended with vomiting and great thirst. Retracted limbs, pain, tumefaction and tenderness of the abdomen, with fever and extreme thirst, indicate inflammation, and are unfavorable. In cholera infantum, clear, liquid stoods, rapid, feeble pulse, intense thirst and sunken orbits, are dangerous symptoms, and if attended with cool, livid surface, and dilated pupils, the case is nearly hopeless. If the child sleep with the eyes half open in all the varieties of the disease, it shows extreme prostration, and is dangerous. If restlessness increase, with constant rolling of the head, with frequent sighing, the cerebral difficulty is paramount, and the recovery need not be expected. This last condition frequently supervenes upon the cessation or abatement of the diarrhœa, and from sympathy with the bowels, through reflex nervous action. It has been considered as metastasis to the brain, but, more probably, is an extension of the disease. The reverse of all these symptoms is favorable.

*Treatment.* While treating of the etiology and pathology of the disease under consideration, I made no allusion to the liver. Recent and extensive investigations, both clinical and autopsical, by Smith, of N. Y., and other able pathologists, have failed to find any structural lesion, functional torpidity, or hypersecretion of that organ, sustaining a causative relation to diarrhœa, in any of its varieties. Hence, "alterative" treatment, designed to "regulate the hepatic secretion," is not only unphilosophical, but may be positively hurtful. Mercury, for ages, has been chiefly relied upon for this purpose. My belief is about confirmed that mercury does not influence, directly, the secretion of the liver. Then, to use it to correct a secretion which is not at fault, whilst it would have no influence upon that secretion if it were, would be doubly untherapeutical. It has been claimed for it, that it acts as a sedative upon the irritated or inflamed mucous membranes. To have this effect, as claimed, it would require doses large enough to act as a cathartic. Now, it is well known to produce *liquid* stools, if it act on the bowels. To increase the liquid stools, when they are already too frequent, too thin and too copious, would be equally unphilosophical. For fifteen years I have discarded calomel, blue mass, mercury with chalk, and all the other preparations of that substance, as an "alterative," in any of the forms of diarrhœa under consideration. They are, to my mind, not only not indicated, but are absolutely injurious. The same is true of all the other so-called alteratives, which have a laxative action.

In all the forms of diarrhœa, if the stomach and bowels are not free from undigested food, or scybala, a laxative is indicated, and when used early, may be all the medical treatment required. Castor oil acts well, and is safe; being partly digested if it does not operate. But in most cases I prefer one that will neutralize acidity—especially if undigested food is the exciting cause. In the milder forms, where no laxative is required, or after its operation, bi-carb. of soda, with peppermint in syrup of anise, may be sufficient, to which paregoric may be added, if deemed necessary. Should it not yield in a day or so, sub-nitrate of bismuth, in four or five grain doses, to a child three to twelve months old, with phosphate of lime if there is nausea or vomiting—given after each stool, or every two to four hours, will usually answer the indications. I have not tried the bromide of potassium, as recently recommended, excepting when there were also convulsions.

In the severer forms, with frequent stools, green, dark, more or less liquid, containing undigested food, condensed casein from the milk, and transparent or tough, opaque mucus, etc., attended with restlessness, thirst and fever, there is congestion of the mucous membrane of the ilium and colon, and if of long standing, inflammation, with the raised glands, etc. Here the indications to be met are, to quiet nervous irritability, check the diarrhœa, and abate the fever. The first and second of these indications may be met by the two following prescriptions, premising, always, that the alimentary canal is free from all offending substances:

| | | |
|---|---|---|
| R. | Vinum Opii, - - - - - - | gtt. xvj. |
| | Ess. Menth. Pip., - - - - | gtt. xl. |
| | Tinct. Catechu, - - - - - | dr. ij. |
| | Sodæ Bi-Carb., - - - - - | gr. xvj. |
| | Syrup Rhei. Arom., - - - - - | dr. iss. |
| | Syrup Anisi, vel. Simplicis, - - - | q. s. |
| M. | Ft. Sol. ad., - - - - - - - | oz. ij. |
| | Sig. A teaspoonful after each stool. | |

| | | |
|---|---|---|
| R. | Bismuthi Sub-Nit., - - - - | dr. iss. |
| | Mucilag. Acaciæ, - - - - | oz. ij. |
| M. | Sig. Shake well, and give a teaspoonful every four hours. | |

These two mixtures may be given as directed, alternating them as to time. But if there be nausea and vomiting, the former may be omitted, and calcis phosph., gr. ij., added to each dose of the other, and given every two or three hours, with sinapism to the epigastrium, until the nausea abates, when the other mixture may be resumed. For the relief of the fever, if considerable, with much tenderness of the bowels, aconite, or veratrum, with ipecac and bi-carbonate soda, may be used. In this last case, sinapisms or turpentine over the abdomen to produce redness, to be followed by simple cataplasms, or flannel cloths wrung from hot water, or hop or other herb tea. If the bowels are known to contain undigested food or scybala, and the stomach will retain no laxative, enemata may be tried. If all else fail to operate, calomel is more apt to be retained, and may act well as *physic*, but not as an *alterative.* I never use it, however, and think my cases are better treated without it.

After the bowels are somewhat checked, and the nausea is relieved, and the fever reduced to a remission or an intermission, in all malarious or mountain localities it is of the first importance to give quinine in liberal doses, in combination with Dover's powder, or its equivalent in opium and ipecac. To a child one year old, three-fourths of a grain to one grain of each of the two first named, every three or four hours—continuing such other treatment as may be necessary. To conceal the taste of quinine, it may be combined with tannic acid and powdered extract of liquorice, given in powder; or the same in syrup of liquorice extract and anise. Many cases readily curable with the use of quinine, are scarcely curable without it. Its action seems to be, to invigorate the entire capillary system, and thus to sustain the healthy functions of all the organs, thereby preserving, unimpaired to the whole system, the full force and power of the vis medicatrix naturæ, on which we chiefly depend, after all, for the recovery. With this view of the *modus operandi*, its great usefulness may be extended to a large range of cases, in all other countries as well as in the malarious or mountain districts.

In cholera infantum, that the fluids of the blood are exhausted—drained off through the bowels—is evinced by the watery stools, the shrunken livid surface, the choked capillaries and the sunken orbits. To the above treatment vigorously pursued, should be added alcoholic stimulants, ammonia, or compound spirits of ether, or other stimulants, with beef or chicken tea in small quantities and often. If the head be unnaturally warm, cold is indicated. To the extremities dry artificial heat should be applied early, and continued. Ice may be given for the thirst.

In *dysentery*, with tormina, tenesmus, and frequent mucous and bloody stools, the colon and rectum are chiefly affected. To the treatment for the severer forms of diarrhœa, above recommended, may be added opiate and astringent enemata, with speedy relief. The menstruum should not exceed one or two teaspoonfuls, and the injection should be retained, if necessary, by supporting the anus with a napkin. These should be repeated, in severe cases, after each stool, until relieved; or if they be forced out immediately, as is often the case, three or four ounces of a solution of nitrate of silver, in flax seed tea, a grain to the ounce, or less, with laudanum, may be injected as far up the rectum as possible with safety, and allowed to return. This is especially useful in chronic cases. Ipecac, in large doses, is recommended in dysentery.

I have given but a brief outline of the treatment, which, however, is usually successful. It would trespass too much on your valuable time to allude farther to the extended therapeutics useful in these cases. Warm baths, friction, sulphuric acid, creosote, carbolic acid, and other vegetable and mineral astringents, and varied enemata, etc., might be dwelt upon. But the treatment herein recommended can be omitted, added to, or changed, to suit special indications. I wish to say, however, that my chief reliance is upon alkalies, bismuth in large and often repeated doses, opiates and quinine; with carminatives, stimulants and local applications at discretion.

Admitting the general pathological views herein expressed, it is obvious that rational prophylaxis and hygiene, with proper regimen in health and during the disease, would save more infantile lives than all medication combined. The subject of *regimen*, in this disease, including diet and clothing, is worthy an entire paper. I beg to say, that there is no diet uniformly appropriate. It must be varied in different cases. For young infants, the mother's milk is the best; except when changed by her menstruation, or pregnancy. If that fail on any account, a healthy wet nurse should be procured if possible.

I have often found in the diarrhœa of infants, that a thin proridge of *groats*, or oat flour made with cream and water, sweetened with pure sugar, to which bi-carbornate of soda is added if the stools are green, will seem to save life. In other cases, soda cracker simmered in a new tin vessel until it becomes gelatinous, and sweetened, may be fed to the infant with the happiest results. Where there are nausea and vomiting, no diet has answered so well in my observations, as chicken tea—made from the flesh of the fowl—free from the skin and fat. It should be rich in the elements of the meat, and seasoned to the taste. It is nearly always relished. Beef or mutton tea, is nearly equal in its effects. For valuable formulæ for infantile diet, see Professor Smith's work on diseases of children.

Great attention should be given to the clothing. It is important to keep the body and limbs well protected with flannel. Although my patients are small, the subject of their ailments is large.

"Great men from little babies grow."

—*Pacific Journal.*

